# In vivo estimation of the shoulder joint center of rotation using magneto-inertial sensors: MRI-based accuracy and repeatability assessment

**DOI:** 10.1186/s12938-017-0324-0

**Published:** 2017-03-21

**Authors:** M. Crabolu, D. Pani, L. Raffo, M. Conti, P. Crivelli, A. Cereatti

**Affiliations:** 1 Department of Electrical and Electronic Engineering, University of Cagliari, Piazza d’Armi, 09123 Cagliari, Italy; 2 Department POLCOMING, University of Sassari, Sassari, Italy; 3 Interuniversity Centre of Bioengineering of the Human Neuromusculoskeletal System, Sassari, Italy; 4 Department of Electronics and Telecommunications, Politecnico di Torino, Turin, Italy

**Keywords:** Human movement, Center of rotation, Magneto-inertial sensing, Accelerometers, Gyroscope, Wearable devices, Shoulder, Gleno-humeral joint, Functional method

## Abstract

**Background:**

The human gleno-humeral joint is normally represented as a spherical hinge and its center of rotation is used to construct humerus anatomical axes and as reduction point for the computation of the internal joint moments. The position of the gleno-humeral joint center (GHJC) can be estimated by recording ad hoc shoulder joint movement following a functional approach. In the last years, extensive research has been conducted to improve GHJC estimate as obtained from positioning systems such as stereo-photogrammetry or electromagnetic tracking. Conversely, despite the growing interest for wearable technologies in the field of human movement analysis, no studies investigated the problem of GHJC estimation using miniaturized magneto-inertial measurement units (MIMUs). The aim of this study was to evaluate both accuracy and precision of the GHJC estimation as obtained using a MIMU-based methodology and a functional approach.

**Methods:**

Five different functional methods were implemented and comparatively assessed under different experimental conditions (two types of shoulder motions: *cross* and *star* type motion; two joint velocities: ω_max_ = 90°/s, 180°/s; two ranges of motion: Ɵ = 45°, 90°). Validation was conducted on five healthy subjects and true GHJC locations were obtained using magnetic resonance imaging.

**Results:**

The best performing methods (NAP and SAC) showed an accuracy in the estimate of the GHJC between 20.6 and 21.9 mm and repeatability values between 9.4 and 10.4 mm. Methods performance did not show significant differences for the type of arm motion analyzed or a reduction of the arm angular velocity (180°/s and 90°/s). In addition, a reduction of the joint range of motion (90° and 45°) did not seem to influence significantly the GHJC position estimate except in a few subject-method combinations.

**Conclusions:**

MIMU-based functional methods can be used to estimate the GHJC position in vivo with errors of the same order of magnitude than those obtained using traditionally stereo-photogrammetric techniques. The methodology proposed seemed to be robust under different experimental conditions. The present paper was awarded as “SIAMOC Best Methodological Paper 2016”.

## Background

An accurate estimation of the gleno-humeral joint center (GHJC) is of primary importance in biomechanics for the upper limb motion analysis [1, 2], the design of robotic arm exoskeletons for shoulder rehabilitation [3], surgical navigation procedures and bones alignment during surgery [4]. The GHJC is commonly employed for defining the humerus anatomical coordinate system [5] and to compute internal joint moments [6]. Being an internal anatomical landmark (AL), the GHJC cannot be identified by palpation as usually done for superficial ALs [7, 8]. The GHJC position is commonly estimated in vivo by using either regressive [7, 9, 10] or functional methods [11–13]. Regressive methods estimate GHJC position from empirical geometrical relations between specific ALs. Functional methods require the subject to perform ad hoc joint movements while recording the motion of the adjacent segments [8]. The assumption underlying these methods is that the gleno-humeral joint (GHJ) is well described by a ball-and-socket joint [14, 15] and its center of rotation (CoR) can be made to coincide with the geometrical center of the medial-superior portion of the humeral head [16].

Several studies have been proposed to estimate the GHJC position using stereo-photogrammetry, electromagnetic or ultrasound positioning systems [7, 9, 11]. These measurement technologies are used to track the three-dimensional positions of selected active or passive markers/sensors attached on the subject’s body. In particular, stereo-photogrammetry marker-based methods have been extensively investigated in terms of formal description [17, 18], using mathematical simulations [19], through experiments using mechanical devices [19, 20], ex vivo [21] and in vivo [22] experiments. While few studies have estimated the precision associated to the GHJC identification in vivo [7, 9, 11], only three works evaluated the accuracy in vivo using medical imaging techniques as gold standard [10, 12, 13]. Campell et al. [10] compared the accuracy and reliability of eight regressive methods, Lampereu et al. [12] compared five different functional methods and Nikooyan et al. [13] tested two functional methods in patients with shoulder hemiarthroplasty.

However, methods based on the use of multiple-camera setup suffer from usability problems in clinical settings and in tele-rehabilitation scenarios. In fact, they require the adoption of expensive equipment, an adequate space for acquisitions, rarely available in the clinical facilities or home-based environment. Furthermore, the expertise of the operator is fundamental for the marker placement, data acquisition and processing. In addition, optical occlusion problems may occur, especially during passive exercises.

The above listed problems could be potentially solved by using miniaturized magneto-inertial measurement units (MIMUs). Sensors wearability, along with the absence of complex external set-up and occlusion problems, make this technology a promising alternative to optical-based approaches. Differently from the stereo-photogrammetry or alternative positioning systems, MIMUs do not provide reliable position measurements, but only accelerations, angular velocities and magnetic field intensity [23]. The measured quantities can be then combined by using sensor-fusion algorithms to obtain MIMU orientation [23].

Previous studies demonstrated that it is possible to estimate the CoR position of a spherical joint using inertial sensors [24–26]. However, the methods validation was carried out on a mechanical analogue of a generic spherical human joint by manually moving a segment with respect to a stationary frame. The abovementioned methods assumed that a unique stationary CoR exists during the functional calibration movement. Conversely, Seel et al. [27] presented a method for the functional estimation of joint axes direction and CoR position, in hinge-like and ball-and-socket-like joints, respectively, which does not require the existence of a stationary CoR. They applied the method for the estimation of the hip joint CoR from data recorded during gait. However, no quantitative information about the errors in the CoR identification was reported. To the best of our knowledge, no studies have been presented for the in vivo functional identification of the GHJC position using MIMU technology.

The aim of this study is to evaluate both accuracy and precision of the GHJC estimation using MIMUs and following a functional approach. In vivo magnetic resonance imaging (MRI) was used as a gold standard for the validation of the methodology on five healthy subjects. Different experimental conditions were considered (joint motion type, movement speed and range of motion, ROM) and five different functional GHJC estimation methods were comparatively evaluated. Selected methods differed for the number of MIMU employed and the algorithms implementation.

## Methods

The following algorithms were implemented and compared.

- The original algorithm proposed by Crabolu et al. [25, 26], based on the use of a single MIMU, hereafter named *Null Acceleration Point* (${\text{NAP}}^{\left( 1 \right)}$), where the superscript indicates the number of MIMUs used;
- A variant of the algorithm ${\text{NAP}}^{\left( 1 \right)}$, which includes a criterion based on the analysis of the angular velocity for selecting the samples to be used for the estimation of the GHJC, hereafter named ${\text{NAP}}_{\upomega}^{\left( 1 \right)}$;
- A variant of algorithm ${\text{NAP}}_{\upomega}^{\left( 1 \right)}$, which uses two MIMUs and exploits the scapulae acceleration information for selecting the samples to be used for the estimation of the GHJC, hereafter named ${\text{NAP}}_{{\upomega {\text{a}}}}^{\left( 2 \right)}$;
- The original algorithm proposed by Seel et al. [27], based on the use of two MIMUs, referred to as Symmetrical Specific Force Center (${\text{SSFC}}^{\left( 2 \right)}$);
- A combination of the two methods 1a and 2, based on the use of two MIMUs, that we named Symmetrical Acceleration Center (${\text{SAC}}^{\left( 2 \right)}$).

All the algorithms exploit rigid body kinematics equations. In this regards, the acceleration of an arbitrary point P of a rigid body *B* during free motion is given by

1 $${\mathbf{a}} = {\mathbf{a}}_{{\mathbf{t}}} + \frac{{d{\varvec{\upomega}}}}{dt} \times {\mathbf{r}} + {\varvec{\upomega}} \times \left( {{\varvec{\upomega}} \times {\mathbf{r}}} \right),$$

where **a**_**t**_ is the acceleration of a point O on the same body, **r** is the position vector from O to P, **ω** is the angular velocity, and $\frac{{d{\varvec{\upomega}}}}{dt}$ the angular acceleration. When a rigid body is constrained through a ball-and-socket joint, it can only experience pure rotational motion around the CoR. If O coincides with the CoR, **at** becomes null, thus Eq. (1) can be computed as:

2 $${\mathbf{a}} = \frac{{d{\varvec{\upomega}}}}{dt} \times {\mathbf{r}} + {\varvec{\upomega}} \times ({\varvec{\upomega}} \times {\mathbf{r}}) .$$

After some algebraic manipulations, Eq. (2) can be rearranged as:

3 $${\mathbf{K}}({\varvec{\upomega}},{\dot{\varvec{\upomega }}}){\mathbf{r}} = {\mathbf{a}}$$

where **K** is equals to

$$\left[ {\begin{array}{*{20}c} {( - \omega_{y}^{2} - \omega_{z}^{2} )} & {(\omega_{x} \omega_{y} - \dot{\omega }_{z} )} & {(\dot{\omega }_{y} + \omega_{x} \omega_{z} )} \\ {(\dot{\omega }_{z} + \omega_{x} \omega_{y} )} & {( - \omega_{x}^{2} - \omega_{z}^{2} )} & {(\omega_{y} \omega_{z} - \dot{\omega }_{x} )} \\ {(\omega_{x} \omega_{z} - \dot{\omega }_{y} )} & {(\dot{\omega }_{x} + \omega_{y} \omega_{z} )} & {( - \omega_{x}^{2} - \omega_{y}^{2} )} \\ \end{array} } \right].$$

Equation (3) is linear with respect to the unknown vector **r**, which represents the CoR position.

### Description of the algorithms

#### Algorithm $NAP^{\left( 1 \right)}$

This algorithm requires a single sensor and assumes that the CoR point has a null acceleration. The vector **r** can be estimated according to Eq. (3) by measuring the observable variables **a** and **ω**, which can be obtained from the data recorded by a MIMU attached to the rotating body. At each sampling time, specific force **f**, angular velocity, and magnetic field vectors are measured with respect to the MIMU coordinate system (MCS). The MCS orientation with respect to a global coordinate system (GCS) can be estimated using a Kalman filter from the measures recorded by the triaxial accelerometer, gyroscope, and magnetometer [23]. In particular, MIMU orientation is required to subtract the gravitational acceleration **g** from the specific force **f** to obtain the coordinate acceleration **a** in Eq. (3). Applying Eq. (3) at each of the N sampling time points recorded during a pure rotational motion of the rigid body, a least-squares solution for **r** can be computed.

#### Algorithm $NAP_{\omega }^{\left( 1 \right)}$

It was proved that to reduce SNR associated to angular velocity signals [26], slow calibration movements should be avoided since they lead to worse results compared to fast movements [25, 26]. To take advantage of this previous observation, only data samples characterized by a magnitude of the angular velocity higher than an empirically chosen threshold, equal to 0.5 rad/s, were included in the least-square solution computation.

#### Algorithm $NAP_{{\omega {\text{a}}}}^{\left( 2 \right)}$

To compensate for moderate violations of the assumption of a null acceleration of the GHJC during the shoulder movement, only data samples for which the magnitude of the acceleration vector of the MIMU placed on the “quasi stationary” body segment was lower than an adaptive threshold were included in the least-square solution computation. The threshold was set equal to the mean magnitude of the acceleration vector of the “quasi stationary” segment. To preserve a minimum number of samples for the computation, when the number of selected samples was less than 300, the threshold was iteratively incremented of 0.1 m/s^2^ until this criterion was met. These values were empirically chosen after some preliminary tests in order to obtain the best results while limiting the number of samples. The applied procedure can be described as follows:

1. Set the threshold “γ” to the mean magnitude of the acceleration vectors measured by the MIMU placed on the “quasi stationary” body segment, **a**_**2i**_:

4 $${\upgamma } = \frac{1}{N}\mathop \sum \limits_{{\varvec{i} = 1}}^{\varvec{N}} \left\| {{\mathbf{a}}_{{2\varvec{i}}} } \right\|;$$

2. Retain the M samples *i* for which $\left\| {{\mathbf{a}}_{{2\varvec{i}}} } \right\| < \gamma$;
3. If M < 300, set γ = γ + 0.1 and repeat point 2, else compute the CoR using the selected M samples.

#### Algorithm $SSFC^{(2)}$ [27]

Let us consider two rigid segments connected by a spherical joint and two MIMUs firmly attached to the segments. Then, the specific force of the CoR as recorded by the two MIMUs should have the same magnitude. It implies that the magnitude of the accelerations experienced by the two MIMUs must be equal each other:

5 $$\left\| {\mathop {\mathbf{a}}\nolimits_{1} - \mathop {\mathbf{K}}\nolimits_{1} (\mathop {\varvec{\upomega}}\nolimits_{1} ,\mathop {{\dot{\varvec{\upomega }}}}\nolimits_{1} )\mathop {\mathbf{r}}\nolimits_{1} } \right\| - \left\| {\mathop {\mathbf{a}}\nolimits_{2} - \mathop {\mathbf{K}}\nolimits_{2} (\mathop {\varvec{\upomega}}\nolimits_{2} ,\mathop {{\dot{\varvec{\upomega }}}}\nolimits_{2} )\mathop {\mathbf{r}}\nolimits_{2} } \right\| = 0$$

where the subscripts denote the quantities measured respectively by the two MIMUs.^1^ Calculating the gradients of Eq. (5) leads to:

6 $$\frac{{d\left\| {{\mathbf{a}}_{i} - {\mathbf{K}}_{i} {\mathbf{r}}_{i} } \right\|}}{{d{\mathbf{r}}_{i} }} = - \frac{{( {\mathbf{a}}_{i} - {\mathbf{K}}_{i} {\mathbf{r}}_{i} )^{T} {\mathbf{K}}_{i} }}{{\left\| {{\mathbf{a}}_{i} - {\mathbf{K}}_{i} {\mathbf{r}}_{i} } \right\|}},\quad i = 1,2$$

Then, it is possible to find those **r**_1_ and **r**_2_ that minimize the quantity in Eq. (5) with the Gauss–Newton algorithm as follows:

1. Define and initialize **x** **=** [**r**_1_, **r**_2_]^T^;
2. Calculate the error vector **e** defined by:

7 $${\mathbf{e}}(n) = \left\| {\mathop {\mathbf{a}}\nolimits_{1} (n) - \mathop {\mathbf{K}}\nolimits_{1} (n)\mathop {\mathbf{r}}\nolimits_{1} } \right\| - \left\| {\mathop {\mathbf{a}}\nolimits_{2} (n) - \mathop {\mathbf{K}}\nolimits_{2} (n)\mathop {\mathbf{r}}\nolimits_{2} } \right\|,\quad n = 1, \ldots ,{\text{N}}$$

3. Use Eq. (6) to calculate the Jacobian (J) d**e**/d**x** and its Moore–Penrose-pseudoinverse pinv(J);
4. Update x by: ${\mathbf{x}} = {\mathbf{x}} - pinv({\mathbf{J}})$ and repeat from step 1.

The vector **x** was initialized to [0 0 0 0 0 0]^T^. The algorithm converged for all the trials analysed in 30 iterations. Convergence was assumed as the achievement of a stable condition in which the solution improvement is < 0.01 m/s^2^.

#### Algorithm $SAC^{\left( 2 \right)}$

If both segments move during the functional movement, the CoR could experience a coordinate acceleration different from zero. However, by rigidly attaching a MIMU on each segment, it is possible to estimate the position of the relative CoR by considering its acceleration **a**_**t**_. Taking in account **a**_**t**_, Eq. (3) becomes:

8 $${\mathbf{K}}({\varvec{\upomega}},{\dot{\varvec{\upomega }}}){\mathbf{r}} + {\mathbf{a}}_{{\mathbf{t}}} = {\mathbf{a}}$$

By computing Eq. (8) for both segments, and substituting **a**_**t**_, it yields:

9 $$\mathop {\mathbf{K}}\nolimits_{1} \mathop {\mathbf{r}}\nolimits_{1} - \mathop {\mathbf{R}}\nolimits_{2}^{1} \mathop {\mathbf{K}}\nolimits_{2} \mathop {\mathbf{r}}\nolimits_{2} = \mathop {\mathbf{a}}\nolimits_{1} - \mathop {\mathbf{R}}\nolimits_{2}^{1} \mathop {\mathbf{a}}\nolimits_{2}$$

where $\mathop {\mathbf{R}}\nolimits_{2}^{1}$ is the rotation matrix from MCS_2_ to MCS_1_.

Equation (9) represents an indeterminate linear system with six unknowns and three equations, but applying Eq. (9) at each of the N sampling instants recorded during the joint movement, an oversized linear system is obtained:

10 $$\left[ {\begin{array}{*{20}c} {{\mathbf{K}}_{1} (1)} & { - {\mathbf{R}}_{2}^{1} (1){\mathbf{K}}_{2} (1)} \\ \vdots & \vdots \\ {{\mathbf{K}}_{1} (N)} & { - {\mathbf{R}}_{2}^{1} (N){\mathbf{K}}_{2} (N)} \\ \end{array} } \right]\left[ {\begin{array}{*{20}c} {{\mathbf{r}}_{1} } \\ {{\mathbf{r}}_{2} } \\ \end{array} } \right] = \left[ {\begin{array}{*{20}c} {{\mathbf{a}}_{1} (1) - {\mathbf{R}}_{2}^{1} (1){\mathbf{a}}_{2} (1)} \\ \vdots \\ {{\mathbf{a}}_{1} (N) - {\mathbf{R}}_{2}^{1} (N){\mathbf{a}}_{2} (N)} \\ \end{array} } \right],$$

and a least-squares solution for **r**_**1**_ and **r**_**2**_ can be computed.

### Experimental design

#### Population

Five subjects (3 M and 2 F) without upper limb disorders were enrolled in the study. The study has been performed following the principles outlined in the Helsinki Declaration of 1975, as revised in 2000, on healthy subjects. The volunteers’ age, height, and body mass index (BMI) were respectively: 36 ± 4 years, 1.7 ± 0.1 m, and 20.7 ± 1.7 kg/m^2^. All participants signed an informed consent form prior to start the recordings, after being informed about the aims and procedures of the experiments. Each subject first underwent an MRI examination and, immediately after, followed a protocol for the evaluation of the GHJC based on a functional approach.

#### Measurement of the GHJC reference position using MRI

**Fig. 1:**
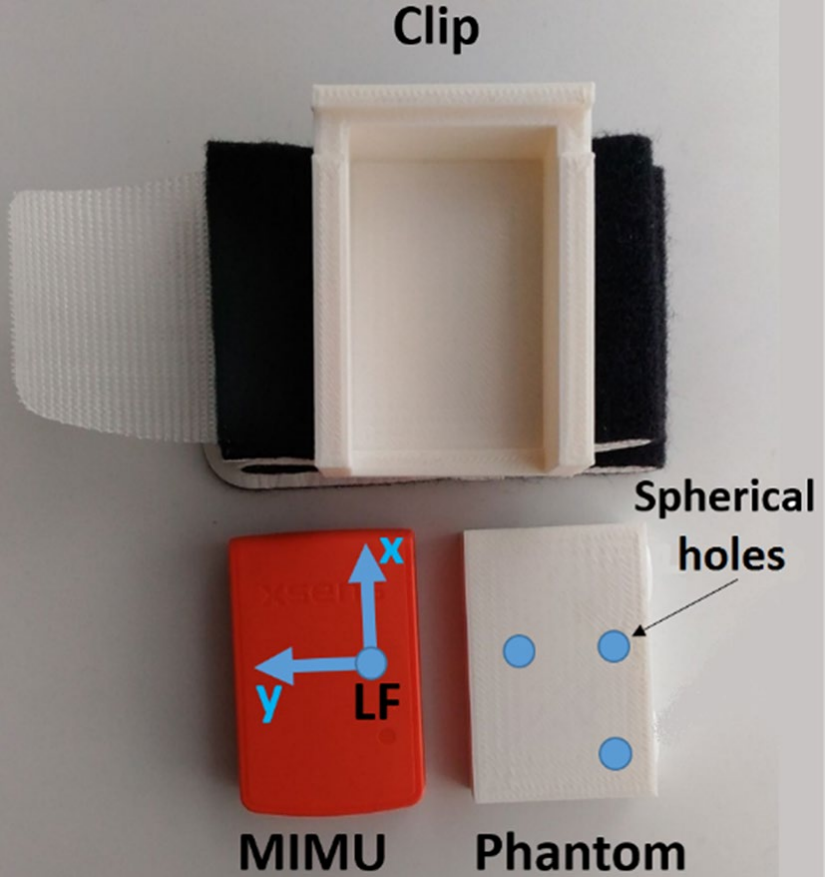
The MIMU, its phantom and the custom clip

**Fig. 2:**
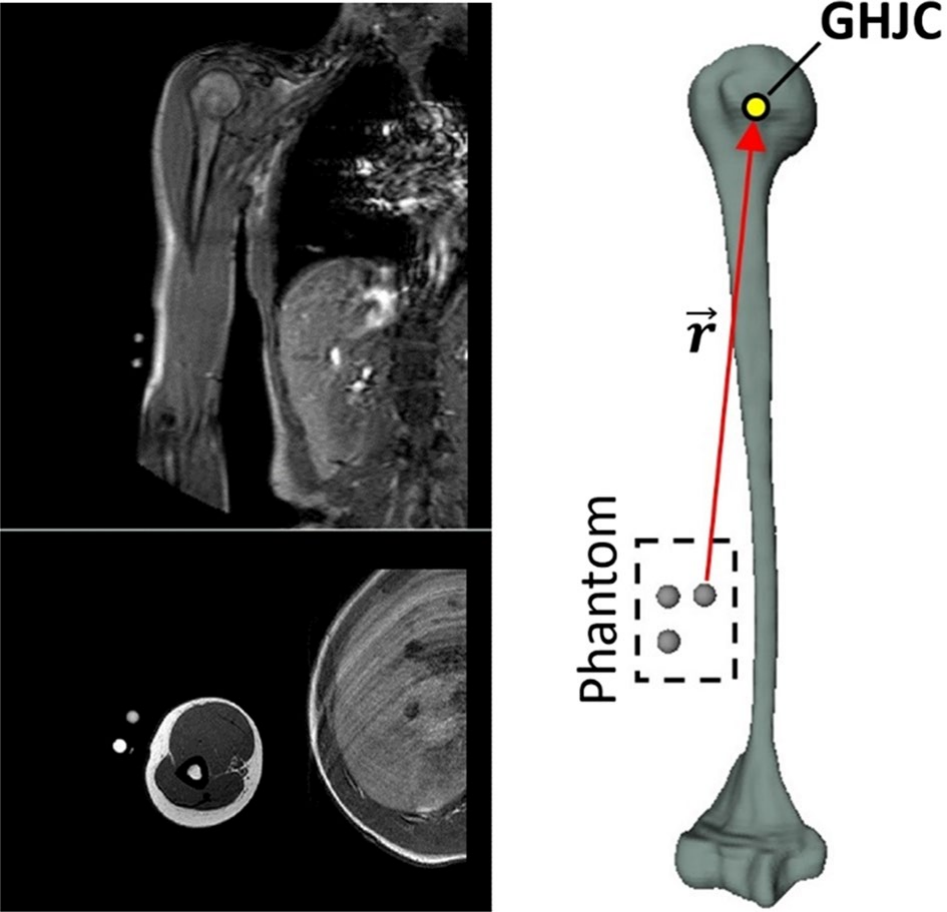
MRI-based humerus and phantom position reconstruction

The MIMU-based GHJC position estimate is expressed with respect to the MCS [26], which is aligned to the edges of the MIMU housing. In order to obtain the gold standard position of the GHJC with respect to the MCS using MRI, a non-ferromagnetic phantom of the MIMU was designed (Fig. 1), and realized by 3D printing (MakerBot Replicator X2) in acrilonitrile–butadiene–stirene (ABS), with a tolerance of 0.1 mm. The phantom included three non-aligned spherical holes filled with copper sulfate in order to be MRI visible, in positions enabling the reconstruction of a local frame coincident to the MCS of the actual MIMU (Fig. 2). One of the three holes was in the same 3D position of the origin of the triaxial accelerometer, with respect to the housing edges. The MIMU, or its phantom, could be fixed to the arm by means of a 3D-printed custom clip and an elastic band.

**Fig. 3:**
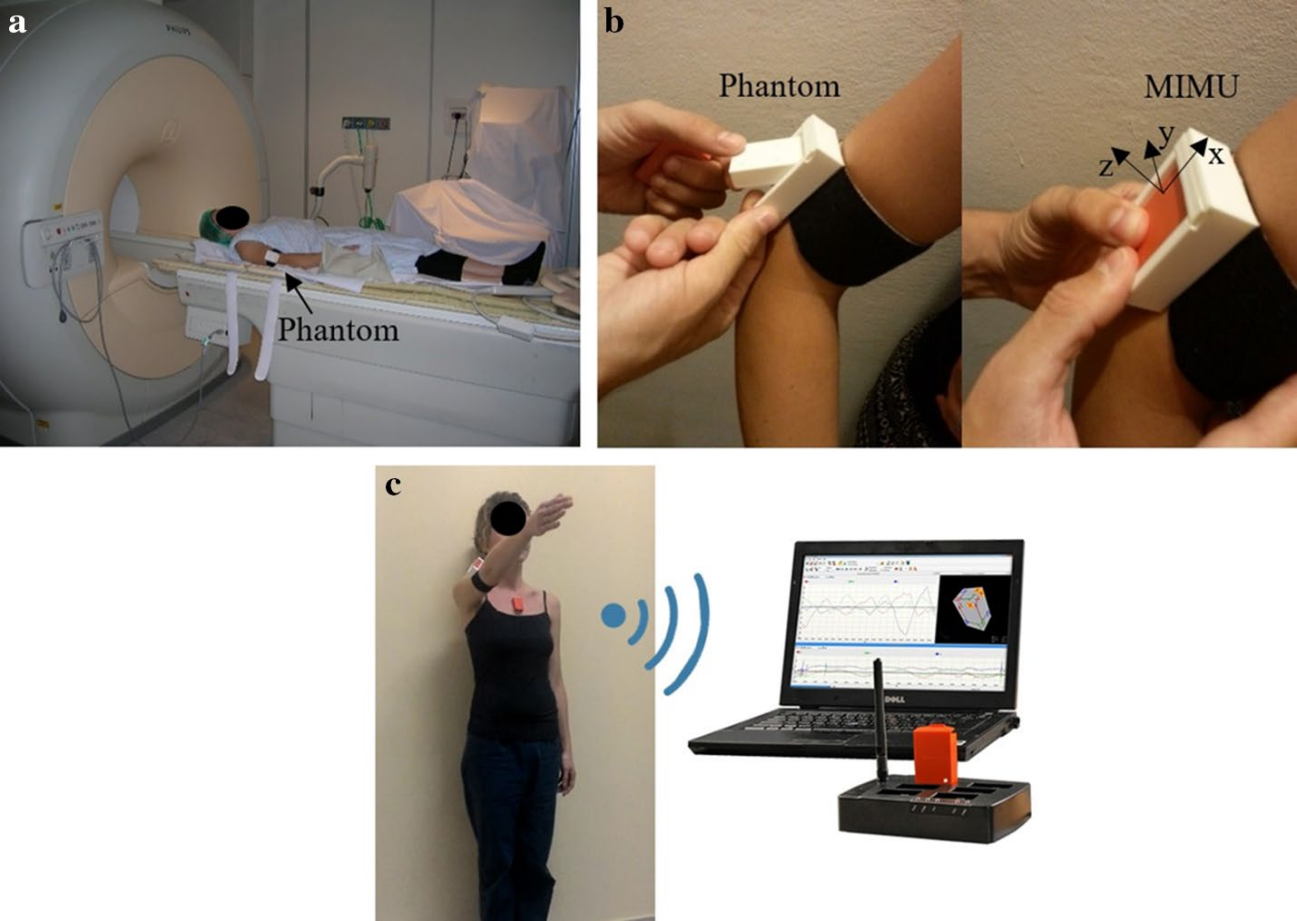
Experiment phases: **a** Scapula, humerus and phantom MRI acquisition; **b** replacing phantom with MIMU. The MIMU was attached laterally to the third distal portion of the arm. With the arm in anatomical position, the MIMU was mounted with *x axis* approximately directed superiorly along the long axis of the humerus, the z axis pointing laterally and the the *y axis* posteriorly; **c** subject executing a shoulder movement, while data were recorded by MIMUs, for the evaluation of the GHJC by functional methods

The MRI data of subjects’ right scapula and humerus were acquired while the phantom was attached to the arm (Fig. 3a). MR scans of the whole right humerus and scapula were obtained by using a 1.5 T MR scanner (Philips Intera Achieva version 1.7). Spin Echo imaging sequences were used (axial T1-W: TR 660 ms; TE 18 ms; flip angle 90 deg; Contiguous Slice Thickness 4 mm, FoV 280 mm). Bone contours were identified using a semiautomatic segmentation procedure. 3D reconstructions of the entire scapular and humeral bones were obtained using the AMIRA image processing software (Visualization Sciences Group, v.5.4). The gold standard GHJC position was estimated as the center of the best fitting sphere to the humeral head of the reconstructed humeral bone [16] (Fig. 2).

Immediately following the MRI acquisition, the phantom was replaced by the actual MIMU, paying attention to preserve the position of the clip (Fig. 3b). In this way, the MCS of the MIMU coincided with the coordinate system of the MR-visible phantom.

#### Experimental protocol

The MIMUs was attached at approximately the same anatomical location for all subjects. A first MIMU was attached laterally to the third distal portion of the arm through the clip and a Velcro strap as shown in Fig. 3. With the arm in anatomical position, the MIMU was mounted with x axis approximately directed superiorly along the long axis of the humerus, the z axis pointing laterally and the y axis posteriorly (Fig. 3). A second MIMU was attached on the scapula with the lower edge along the cranial edge of the spina scapulae using a double-sided tape [28]. A third MIMU was fixed on the thorax at the sternum level. This MIMU was not used to compute the GHJC but only to monitor the amplitude of the thorax motions during the functional movement. Each MIMU comprises triaxial accelerometer, gyroscope and magnetometer (MTw2 Awinda wireless motion tracker system, Xsens, sample frequency: 100 Hz; dynamic accuracy: Roll/pitch = 0.75° RMS; Heading = 1.5° RMS). The sensor position with respect to the housing was provided by the manufacturer. A 15-min warm-up period was included before starting the data collection. A preliminary spot-check of the MIMUs orientation estimates was performed according to the guidelines proposed in [29]. Briefly, the MIMUs were aligned to each other and attached to a rigid plastic plate; then, the differences in the orientation estimates provided by each MIMU were computed while the orientation of the plastic plate was being manually varied about the three directions. This dynamic test had a duration of 30 s. In addition, the gyroscope bias and the residual acceleration after compensation of the gravity during a 30 s static recording were computed.

Subjects were instructed to perform arm movements trying to minimize the movement of the trunk and were allowed to practise few times before starting the recordings (Fig. 3c). Data were recorded under six different experimental conditions: two types of shoulder motions (*cross* and *star* type motion as in Table 1), two joint velocities (*fast* movements: ω_max_ = 180°/s, *slow* movements: ω_max_ = 90°/s) and two ranges of motion (Ɵ_1_ = 45°; Ɵ_2_ = 90°). Each trial comprised a static acquisition (5 s) followed by the selected shoulder motion. Three repetitions for each condition were recorded, for a total of 24 trials per subject.

**Table 1:** Description of the two different types of joint movements

Motion type	Graphical representation	Description
Cross		With the elbow joint in maximum extension, the hand describes two arcs generated by thoraco-humeral rotations consisting in two successive elevation movements (ROM equals to Ɵ; plane of elevation = 0°: abduction, 90°: forward flexion)
Star		With the elbow joint in maximum extension, the hand describes four arcs generated by thoraco-humeral rotations consisting in four successive elevation movements (ROM equals to Ɵ; plane of elevation = 0°: abduction, 30°, 60°, 90°: forward flexion)

Acquisitions were performed on each subject by the same examiner.

### Data processing and analysis

For each MIMU, the bias affecting the gyroscopes during the joint motion was partially compensated by subtracting the mean angular velocity value obtained during the spot-check. Prior to the differentiation, the angular velocity was filtered using a decimated wavelet denoising approach exploiting the Bior3.3 mother wavelet and soft-thresholding. Fixed level-dependent thresholds over the chosen four decomposition levels were used, whereas level 1 was completely cleared out from the signal, achieving a low-pass behavior and limiting the signal bandwidth up to a quarter of the sampling frequency. The angular acceleration was computed by differentiating the angular velocity signal with a three-point central difference operator.

For each trial and algorithm, the vector difference ***e***_***i***_ between the gold standard GHJC location and the estimated one, and the scalar difference *e*_*ri*_ between the true radius length (|**r**|) and the estimated one, were computed. For each subject, the overall algorithm accuracy was computed in terms of *E and E*_*r*_ values obtained by averaging the module of ***e***_***i***_ and *e*_*ri*_ over the 24 trials (2 type of motions × 2 two joint velocities × two ranges of motion × 3 trials repetitions), as follows:

11 $$E = \frac{1}{{24}}\sum\limits_{{i = 1}}^{{24}} {\left\| {\varvec{e}_{i} } \right\|}$$

12 $$E_{r} = \frac{1}{24}\mathop \sum \limits_{i = 1}^{24} e_{ri}$$

where $\left\| \cdot \right\|$ is the Euclidean distance.

The repeatability associated to the GHJC estimation was computed for each algorithm in terms of *E*_*SD*_ value:

13 $$E_{SD} = \frac{1}{24}\mathop \sum \limits_{i = 1}^{24} \sqrt {SD_{xi}^{2} + SD_{yi}^{2} + SD_{zi}^{2} }$$

where *SD*_*j*_, with j = x, y or z, is the standard deviation of each estimated GHJC position coordinate.

Preliminary normality test (Shapiro–Wilk test) was performed to choose the most appropriate statistical analysis. For each algorithm, we investigated whether the method’s accuracy was affected by the following independent factors: (a) joint angular velocity, (b) type of the joint motion, (c) joint angular ROM. To this purpose, a two-tailed Student’s t test was performed for each one of the independent factors analysis above (individual subject comparisons).

In order to assess if there was a significant difference between the five algorithms (${\text{NAP}}^{\left( 1 \right)}$, ${\text{NAP}}_{\upomega }^{\left( 1 \right)}$, ${\text{NAP}}_{{\upomega {\text{a}}}}^{\left( 2 \right)}$, ${\text{SSFC}}^{\left( 2 \right)}$, ${\text{SAC}}^{\left( 2 \right)}$), one-way ANOVA test for normal samples distribution was used (individual subject comparisons). When a significant difference was detected (*p* < 0.05), a Student’s t test was performed between every pair of methods.

We also evaluated whether differences in the GHJC location error component along each anatomical direction were present, by performing a Student’s t test for each pair of coordinates (individual subject comparisons).

A Bonferroni’s correction was used when multiple comparisons were studied.

## Results

### MIMU spot check

Results relative to the spot-check performed on the MIMUs attached over the humerus and scapula are shown in Table 2.

**Table 2:** Spot check results

	Orientation difference (degrees)			Bias in angular velocity (°/s)			Residual acceleration (m/s^2^)		
	Roll	Pitch	Yaw	x	y	z	x	y	z
M_h_	0.2 ± 0.1	0.2 ± 0.1	2 ± 0.4	0.23 ± 0.06	0.23 ± 0.11	−0.85 ± 0.11	−0.001 ± 0.02	−0.011 ± 0.01	0.001 ± 0.02
M_s_				−0.17 ± 0.06	0.57 ± 0.51	0.11 ± 0.11	0.002 ± 0.02	0.004 ± 0.04	0.003 ± 0.02

M_h_ and M_s_ refer to the MIMUs attached over the humerus and on the scapulae, respectively

### Effects of joint angular velocity, type of joint motion, joint angular ROM

For each algorithm and each subject, no statistically significant differences were observed between *slow* and *fast* joint angular velocities and between *cross* and *star* joint motion (*p* > 0.05, Student’s t test). Even for the different joint ROM, no statistically significant differences were observed except in the following cases: error *E*_*r*_, subject 2, methods NAP and ${\text{SSFC}}^{\left( 2 \right)}$; error *E,* subjects 4 and 5, method ${\text{SAC}}^{\left( 2 \right)}$ (*p* < 0.05, Student’s t test). For the sake of brevity, the results relative to each independent factor are shown only for the algorithm ${\text{NAP}}_{\upomega }^{\left( 1 \right)}$ (Table 3).

**Table 3:** *E* and *E*_*r*_ (mm) for each subject and each experimental factor for the ${\text{NAP}}_{\upomega}^{\left( 1 \right)}$ algorithm

Factor	Subject 1		Subject 2		Subject 3		Subject 4		Subject 5		Mean	
	*E*	*E*_*r*_	*E*	*E*_*r*_	*E*	*E*_*r*_	*E*	*E*_*r*_	*E*	*E*_*r*_	*E*	*E*_*r*_
Slow	14.8 ± 5	9.8 ± 7	34.6 ± 6	13.8 ± 7	11.5 ± 5	6.3 ± 2	21.2 ± 2	4.7 ± 2	15.5 ± 6	11.2 ± 7	19.5 ± 10	9.1 ± 6
Fast	16.9 ± 9	7.4 ± 5	40.4 ± 11	11.5 ± 10	11.4 ± 4	9.6 ± 4	21.1 ± 3	3.6 ± 2	19.1 ± 4	9.2 ± 5	21.8 ± 12	8.3 ± 6
Cross	17.6 ± 6	7.9 ± 7	37.6 ± 11	12.7 ± 9	11.4 ± 5	7.9 ± 3	22.6 ± 3	4.5 ± 2	17.6 ± 6	8.2 ± 7	21.4 ± 11	8.2 ± 6
Star	14.1 ± 8	9.3 ± 5	37.4 ± 8	12.6 ± 9	11.4 ± 4	8.0 ± 4	19.7 ± 2	3.8 ± 2	16.9 ± 4	12.2 ± 5	19.9 ± 11	9.2 ± 6
Ɵ_*1*_	18.6 ± 8	7.2 ± 5	36.5 ± 7	16.5 ± 9	9.6 ± 5	8.1 ± 4	20.0 ± 2	3.8 ± 2	16.1 ± 6	8.6 ± 5	20.2 ± 11	8.8 ± 7
Ɵ_*2*_	13.2 ± 6	9.9 ± 7	38.5 ± 11	8.8 ± 6*	13.2 ± 4	7.8 ± 3	22.3 ± 3	4.5 ± 2	18.5 ± 3	11.8 ± 7	21.1 ± 11	8.6 ± 6

Mean ± STD

* A statistically significant difference between the relevant parameters (*p* < 0.05, Student’s t test)

### Accuracy and repeatability of the tested algorithms

**Fig. 4:**
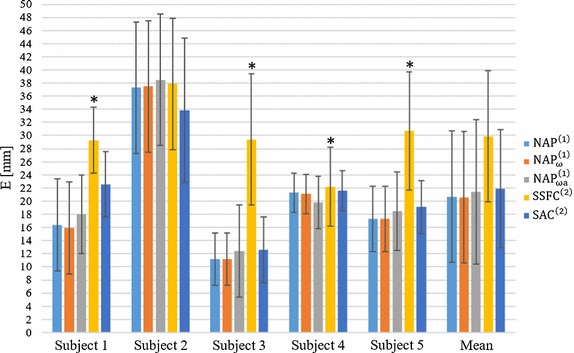
Bar chart of the Euclidean distance (*E*) for each subject and each algorithm between the true GHJC and the estimated one. A *star* indicates a statistically significant difference between that specific algorithm (on that subject) and all the other algorithms

The values of *E* (mean and standard deviation, *STD*), obtained for the different five algorithms for each subject, are reported in Table 4 and in the bar chart of Fig. 4. The smallest mean *E* value was obtained with the ${\text{NAP}}_{\upomega }^{\left( 1 \right)}$ algorithm (20.6 ± 10 mm). With this algorithm, E over each subject ranges between a minimum of 11.2 mm (subject 3) and 37.5 mm (subject 2). The lowest accuracy was found with the ${\text{SSFC}}^{\left( 2 \right)}$ algorithm (29.9 ± 10 mm, ranging across subjects from 22.2 to 37.9 mm).

**Table 4:** Accuracy errors *E* in mm for each subject and each algorithm between the true GHJC and the estimated one

Algorithm	Subject 1	Subject 2	Subject 3	Subject 4	Subject 5	Mean
${\text{NAP}}^{\left( 1 \right)}$	16.4 ± 7	37.3 ± 10	11.2 ± 4	21.3 ± 3	17.3 ± 5	20.7 ± 10
${\text{NAP}}_{{\upomega}}^{\left( 1 \right)}$	15.9 ± 7	37.5 ± 10	11.2 ± 4	21.1 ± 3	17.3 ± 5	20.6 ± 10
${\text{NAP}}_{{\upomega {\text{a}}}}^{\left( 2 \right)}$	18.0 ± 6	38.5 ± 10	12.4 ± 7	19.8 ± 4	18.5 ± 6	21.4 ± 11
${\text{SSFC}}^{\left( 2 \right)}$	29.3 ± 5*	37.9 ± 10	29.4 ± 10*	22.2 ± 6*	30.7 ± 9*	29.9 ± 10
${\text{SAC}}^{\left( 2 \right)}$	22.6 ± 5	33.8 ± 11	12.6 ± 5	21.6 ± 3	19.1 ± 4	21.9 ± 9

Mean ± STD. A star indicates a statistically significant difference between that specific algorithm (on that subject) and all the other algorithms, with p < 0.005 (Student’s t test with Bonferroni’s correction)

**Fig. 5:**
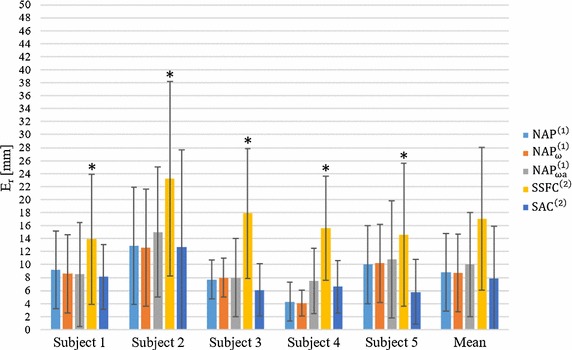
Bar chart of the error *E*_*r*_ for each subject and each algorithm between estimated radius and measured by MRI. A *star* indicates a statistically significant difference between that specific algorithm (on that subject) and all the other algorithms

The values of *E*_*r*_ (mean and *STD*) obtained for each algorithm for each subject are reported in Table 5 and in the bar chart of Fig. 5. Also for the *E*_*r*_, the smallest value was obtained for ${\text{NAP}}_{\upomega}^{\left( 1 \right)}$ algorithm (8.7 ± 6 mm). The minimum value was detected for the subject 4 (4.1 ± 2 mm), whereas the highest value was again obtained on subject 2 (12.6 ± 9 mm). The largest mean *E*_*r*_ was obtained with ${\text{SSFC}}^{\left( 2 \right)}$ algorithm (17.1 ± 11 mm, ranging from 13.9 to 23.2 mm).

**Table 5:** Accuracy error *E*_*r*_ (mean ± STD) in mm for each subject and each algorithm between estimated radius and the actual one measured by MRI

Algorithm	Subject 1	Subject 2	Subject 3	Subject 4	Subject 5	Mean
${\text{NAP}}^{\left( 1 \right)}$	9.2 ± 6	12.9 ± 9	7.7 ± 3	4.3 ± 3	10.0 ± 6	8.81 ± 6
${\text{NAP}}_{\upomega }^{\left( 1 \right)}$	8.6 ± 6	12.6 ± 9	8.0 ± 3	4.1 ± 2	10.2 ± 6	8.7 ± 6
${\text{NAP}}_{{\upomega{\text{a}}}}^{\left( 2 \right)}$	8.5 ± 8	15.0 ± 10	8.0 ± 6	7.5 ± 5	10.8 ± 9	10.0 ± 8
${\text{SSFC}}^{\left( 2 \right)}$	13.9 ± 10*	23.2 ± 15*	17.9 ± 10*	15.6 ± 8*	14.6 ± 11*	17.1 ± 11
${\text{SAC}}^{\left( 2 \right)}$	8.1 ± 5	12.7 ± 15	6.1 ± 4	6.6 ± 4	5.8 ± 5	7.9 ± 8

* A statistically significant difference between that specific algorithm (on that subject) and all the other algorithms, with *p* < 0.005 (Student’s t test with Bonferroni’s correction)

The repeatability results (E_SD_) are displayed for each subject and algorithm in Table 6. The ${\text{NAP}}_{\upomega }^{\left( 1 \right)}$ algorithm showed the smallest E_SD_ mean value compare to the other algorithms (9.4 mm, ranging from 5.3 to 16.3 mm). The ${\text{SSFC}}^{\left( 2 \right)}$ algorithm returned the largest E_SD_ values (13.8 mm, ranging from 9.2 to 17.3 mm).

**Table 6:** Repeatability values E_SD_ in mm on GHJC identification for each subject according to the five algorithms

Algorithm	Subject 1	Subject 2	Subject 3	Subject 4	Subject 5	Mean
${\text{NAP}}^{\left( 1 \right)}$	10.4	16.1	6.9	5.7	8.5	9.5
${\text{NAP}}_{\upomega }^{\left( 1 \right)}$	10.2	16.3	7	5.3	8.4	9.4
${\text{NAP}}_{{\upomega{\text{a}}}}^{\left( 2 \right)}$	11.5	16.4	8.24	6.7	11.5	10.9
${\text{SSFC}}^{\left( 2 \right)}$	14.5	17.3	13.5	9.2	14.3	13.8
${\text{SAC}}^{\left( 2 \right)}$	11	19	7.2	5.8	9.2	10.4

### Algorithms comparison

The ANOVA test showed that the resulting *E* and *E*_*r*_ were significantly different among algorithms. In Tables 4 and 5, the algorithm results are marked with one star when a statistically significant difference was detected between that algorithm (for a given subject) and the others (p < 0.005, Student’s t test with Bonferroni’s correction). A significant difference was observed only between the ${\text{SSFC}}^{\left( 2 \right)}$ and the other algorithms for both *E* and *E*_*r*_ with the exception of *E* values in the subject 2. No significant differences were detected among the other four algorithms (${\text{NAP}}^{\left( 1 \right)}$, ${\text{NAP}}_{\upomega}^{\left( 1 \right)}$, ${\text{NAP}}_{{\upomega{\text{a}}}}^{\left( 2 \right)}$, ${\text{SAC}}^{\left( 2 \right)}$).

### GHJC error directionality

For all subjects and all methods, significant differences were observed between the errors along the x and z directions (marked with a circle in Table 7) as well as x and y except for the subject 5; between y and z directions a significant difference was detected only for the subjects 1 and 5 (p < 0.016, Student’s t test and Bonferroni’s correction). For the sake of brevity, the results along each direction in estimating GHJC are reported only for the algorithm ${\text{NAP}}_{\upomega}^{\left( 1 \right)}$ (Table 7).

**Table 7:** Absolute error (mean ± STD) along each coordinate for each subject using the ${\text{NAP}}_{\upomega }^{\left( 1 \right)}$ algorithm

Coordinate	Subject 1	Subject 2	Subject 3	Subject 4	Subject 5	Mean
x	7.8 ± 5^º,^*	15.0 ± 11^º,^*	8.0 ± 4^º,^*	3.2 ± 3^º,^*	9.5 ± 5^º^	8.7 ± 7
y	10.7 ± 8	22.4 ± 7	4.3 ± 3	15.3 ± 3	11.2 ± 4	12.8 ± 8
z	5.0 ± 3*	22.5 ± 10	4.2 ± 4	13.5 ± 4	5.8 ± 5*	10.3 ± 9

A star and/or a circle in the x row indicate a statistically significant difference respectively from y and/or z directions, while in the z row a star indicates a statistically significant difference from y direction (p < 0.016, Student’s t test with Bonferroni’s correction)

## Discussion

In the present study, we performed an evaluative comparison, in terms of accuracy and repeatability, of five different algorithms for the in vivo identification of the GHJC using magneto-inertial sensing technology. The methods accuracy was evaluated on five healthy subjects, using as gold standard the GHJC positions determined from bio-imaging (MRI).

The analysis performed on the five participants seemed to indicate that the accuracy of the tested methods was not significantly affected by the type of arm motion analyzed (star vs. cross) or the reduction of the arm angular velocity from 180°/s to 90°/s. Similarly, a reduction of the joint ROM from 90° to 45° did not seem to influence significantly the GHJC position estimate except in a few subject-method combinations.

These results seemed to confirm the robustness of the methodology proposed under different experimental conditions. However, when defining the experimental protocol for the functional identification of the GHJC position, good practice guidelines should be followed (slow joint movement should be avoided, and these movements should involve at least two different axes of rotation and allowing acquisition of sufficient number of samples) [26].

The null acceleration point methods (${\text{NAP}}^{\left( 1 \right)}$, ${\text{NAP}}_{\upomega}^{\left( 1 \right)}$ and ${\text{NAP}}_{{\upomega{\text{a}}}}^{\left( 2 \right)}$) and the Symmetrical Acceleration Center ${\text{SAC}}^{\left( 2 \right)}$ method outperformed the Symmetrical Specific Force Center (${\text{SSFC}}^{\left( 2 \right)}$) method. By analysing each of the five participants, we could not find any significant differences among the different NAP method implementations. This would suggest that the sample selection procedures implemented in both ${\text{NAP}}_{\upomega}^{\left( 1 \right)}$ and ${\text{NAP}}_{{\upomega{\text{ a}}}}^{\left( 2 \right)}$ algorithms were not effective under the specific experimental conditions analyzed. However, it cannot be excluded that differences might arise for slower movements and/or larger movement of the trunk segment. Considering the highest repeatability and accuracy of the algorithm ${\text{NAP}}_{\upomega }^{\left( 1 \right)}$ and its advantage in using a single sensor, we suggest the use of this algorithm when estimating the GHJC position in similar experimental conditions.

GHJC position errors computed over all subjects slightly varied among NAP and $SAC^{\left( 2 \right)}$ methods (20.6 to 21.9 mm), whereas repeatability varied between 9.4 and 10.4 mm.

Interestingly, the use of a second MIMU attached to the scapula (${\text{NAP}}_{{\upomega{\text{a}}}}^{\left( 2 \right)}$ and $SAC^{\left( 2 \right)}$) seemed to offer no advantages with respect to single unit methods (${\text{NAP}}^{\left( 1 \right)}$, ${\text{NAP}}_{\upomega }^{\left( 1 \right)}$). This results can be explained by the intrinsic difficulties in tracking the scapular motion due to the soft tissue artefacts [30] and the fact that the trunk movements observed for all subjects during the functional exercises were quite limited (ROM < 30, trunk angular velocity RMS < 30°/s).

The ${\text{SSFC}}^{\left( 2 \right)}$ algorithm was the less accurate and repeatable method despite the fact it does not require to remove the gravity contribution and was conceived to take into account also for a translation of the CoR. An explanation of the poorer performance compared to the other methods can be provided by the small amplitude of the signals recorded by the second MIMU positioned on the scapula (ω_max_ < 30°/s) and the difficulty related to the scapula tracking.

For all methods, errors along the x axis (approximately longitudinal anatomical direction of the humerus) resulted slightly lower than those in the other directions. This might be either due to anisotropy of the soft tissue artefact components or to the shoulder motions performed.

To the best of authors’ knowledge, this is the first study investigating functional methods for the GHJC estimation in vivo using MIMUs, exploiting a medical imaging approach as gold standard. The errors associated to the best performing methods were of the same order of magnitude than those obtained using a functional approach from marker-based stereo-photogrammetric systems [10, 12, 13] and smaller than those found by using the regressive method recommended by the International Society of Biomechanics (ISB) [7] (about 30 mm, on average) [10]. Lampereu et al. [12] obtained on four subjects errors ranging between 11 and 17 mm with five different functional methods. Nikooyan et al. tested the symmetrical CoR estimation (Score) [18] and instantaneous helical axis [15] methods in five patients with shoulder hemiarthroplasty, finding accuracy errors respectively of 20.7 and 14.7 mm [13]. GHJC errors found in the present in vivo study were about five times higher than those found on a mechanical analogue [25, 26]. These differences were expected and they could be mainly ascribed to: (1) the presence of soft tissue artefacts, which can introduce a relative movement between the body and device which is influenced by the mass of the MIMU and by the fixing technique; (2) the fact that the GHJC is not perfectly stationary during the calibration movements potentially affecting methods ${\text{NAP}}^{\left( 1 \right)}$, ${\text{NAP}}_{\upomega }^{\left( 1 \right)}$, ${\text{NAP}}_{{\upomega{\text{a}}}}^{\left( 2 \right)}$. In fact, due to the scapula-humeral rhythm, the scapula remains stationary only for the first 30° of humerus abduction. Furthermore, it is difficult for the subject to completely avoid trunk movements during the calibration exercise.

Interestingly, we found that both in the experiments using a mechanical linkage and a mathematical simulation [24] and the present in vivo study, the type and range of arm motion were not critical. Conversely, while in the study on the mechanical linkage we observed an increase of the errors when the angular velocity was decreased from 180°/s to 60°/s, in the present study we could not find any significant trend. This discrepancy could be partially explained by the larger angular velocity tested in the in vivo experiments (*slow movements* characterized by ω_max_ = 90°/s). However, due to the limited number of subjects analysed (five), caution is required when generalizing the study findings. Future studies including a larger number of participants, with different anthropometric characteristics, from both healthy and pathological populations, are required to further validate the proposed methodologies.

## Conclusions

Based on the study evidences, it is possible to estimate in vivo the GHJC by using magneto-inertial sensors and a functional approach, obtaining accuracy and repeatability of the same order of magnitude of those achievable with more expensive and complex stereo-photogrammetry techniques. Under the different experimental conditions analyzed and in presence of limited trunk movements, GHJC errors of about 20 mm were achieved using a single MIMU. The positioning of an additional MIMU did not improve the methods performance. However, further analyses are needed to investigate the advantage associated to the use of two MIMUs when large trunk movements are observed.

## Abbreviations

GHJC: Gleno-humeral joint center
GHJ: Gleno-humeral joint
MIMUs: magneto-inertial measurement units
AL: anatomical landmark
CoR: center of rotation
ROM: range of motion
MRI: magnetic resonance imaging
NAP: null acceleration point
SSFC: Symmetrical Specific Force Center
SAC: Symmetrical Acceleration Center
MCS: MIMU coordinate system
GCS: global coordinate system
BMI: body mass index
ABS: acrilonitrile–butadiene–stirene
*e*: vector difference *e* between the true GHJC location and the estimated one
*e*_*r*_: scalar difference between the true radius length (|**r**|) and the estimated one
*E*: mean Euclidean distance
*E*_*r*_: mean *e*_*r*_
*E*_*SD*_: repeatability
STD: standard deviation of the error
ISB: International Society of Biomechanics
Score: symmetrical CoR estimation

## Footnotes

1 The accelerations in Eq. (5) (**a**_**1**_, **a**_**2**_) represent the specific forces as they are recorded by the MIMUs. For the sake of clarity, we preserved the same notation.
